# cGMP-Dependent Protein Kinase I Is Crucial for Angiogenesis and Postnatal Vasculogenesis

**DOI:** 10.1371/journal.pone.0004879

**Published:** 2009-03-16

**Authors:** Alexandra Aicher, Christopher Heeschen, Susanne Feil, Franz Hofmann, Michael E. Mendelsohn, Robert Feil, Stefanie Dimmeler

**Affiliations:** 1 Department of Internal Medicine III, J. W. Goethe University, Frankfurt, Germany; 2 Interfakultäres Institut für Biochemie, Universität Tübingen, Tübingen, Germany; 3 Institut für Pharmakologie und Toxikologie, Technische Universität München, München, Germany; 4 Molecular Cardiology Research Institute, Tufts Medical Center, Tufts University School of Medicine, Boston, Massachusetts, United States of America; University of Giessen Lung Center, Germany

## Abstract

**Background:**

Endothelium-derived nitric oxide plays an important role for the bone marrow microenvironment. Since several important effects of nitric oxide are mediated by cGMP-dependent pathways, we investigated the role of the cGMP downstream effector cGMP-dependent protein kinase I (cGKI) on postnatal neovascularization.

**Methodology/Principal Findings:**

In a disc neovascularization model, cGKI^−/−^ mice showed an impaired neovascularization as compared to their wild-type (WT) littermates. Infusion of WT, but not cGKI^−/−^ bone marrow progenitors rescued the impaired ingrowth of new vessels in cGKI-deficient mice. Bone marrow progenitors from cGKI^−/−^ mice showed reduced proliferation and survival rates. In addition, we used cGKIα leucine zipper mutant (LZM) mice as model for cGKI deficiency. LZM mice harbor a mutation in the cGKIα leucine zipper that prevents interaction with downstream signaling molecules. Consistently, LZM mice exhibited reduced numbers of vasculogenic progenitors and impaired neovascularization following hindlimb ischemia compared to WT mice.

**Conclusions/Significance:**

Our findings demonstrate that the cGMP-cGKI pathway is critical for postnatal neovascularization and establish a new role for cGKI in vasculogenesis, which is mediated by bone marrow-derived progenitors.

## Introduction

Mice deficient for endothelial nitric oxide synthase (eNOS) show a defective neovascularization [1], which is, at least in part, related to an impaired mobilization of vasculogenic progenitor cells [2]. In contrast to angiogenesis, which results from the migration and proliferation of pre-existing mature endothelial cells [3], vasculogenesis is based on the recruitment and incorporation of bone marrow-derived progenitors to sites of neovascularization [4]–[6]. Endothelium-derived nitric oxide (NO) plays an essential role in the bone marrow microenvironment and is essential for the mobilization of progenitor cells [2], [7]–[10]. However, NO bioavailability is typically reduced in patients with diabetes, coronary artery disease, and ischemic cardiomyopathy (ICMP) [11]–[13]. Consequently, bone marrow progenitors from patients with diabetes or ICMP show a reduced neovascularization capacity, which is a drawback for their use in cell therapy [14]. The impaired functional activity of bone marrow cells from patients could be partially restored after pharmacological enhancement of eNOS expression or nitric oxide donors, which highlights the important role of endothelium-derived NO for neovascularization [15], [16]. One important downstream signaling pathway of endothelium-derived NO is mediated by cyclic guanosine monophosphate (cGMP) as second messenger. Elevated levels of intracellular cGMP modulate phosphodiesterases, open cation channels, and activate cGMP-dependent protein kinases (cGK; also called protein kinase G; PKG) [17]. The cGKs belong to the family of serine/threonine kinases [18], [19]. Mammals have two cGK genes, which encode cGKI and cGKII. The NH_2_-terminus of cGKI is encoded by two alternative exons producing the isoforms cGKIα and cGKIβ. The cGKI isoforms are soluble enzymes and interact with several proteins through their NH_2_-termini containing leucine/isoleucine zippers (LZ). Here, we investigated the role of the NO downstream signaling protein cGKI for postnatal neovascularization in two different models for cGKI deficiency, cGKI^−/−^ mice and mice with a cGKI mutation that affects downstream signaling (LZM mice).

## Results

**Figure 1:**
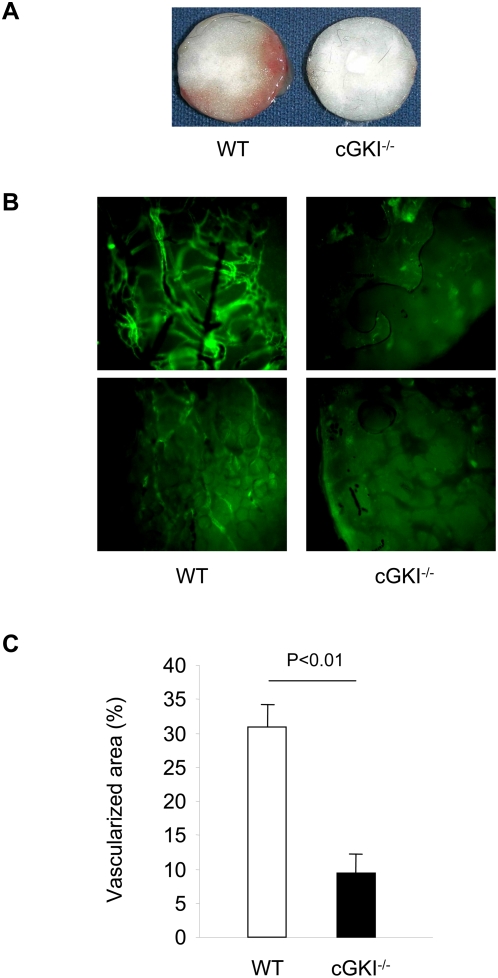
Growth of vessels in the neovascularization disc model of cGKI^−/−^ and wild-type (WT) mice. (A) Discs after explantation from cGKI^−/−^ and WT mice. (B) Vessels growing from the rim of the disc into the center in cGKI^−/−^ and WT mice identified by staining with CD31 (green). (C) Quantification of the vascularized area (n = 10).

To study the role of cGKI for neovascularization *in vivo*, we subcutaneously implanted discs into the flanks of cGKI^−/−^ mice and their wild-type (WT) littermates. The implantation of discs stimulates neovascularization due to sterile inflammation. After two weeks, animals were sacrificed, and, prior to explantation of the disks, fluorescent microspheres were infused into the circulation to demonstrate the perfusion of the vessels growing from the rim to the center of the disc. Gross examination of the explanted disk already revealed a clear reduction in the vascularized area for cGKI^−/−^ mice compared to WT (**Fig. 1A**). In addition, formation of neovessels was demonstrated by staining for the endothelial marker CD31 (**Fig. 1B**). Quantitative analysis demonstrated that neovascularization of the discs in cGKI^−/−^ mice was strongly reduced compared with their WT littermates (**Fig. 1C**).

**Figure 2:**
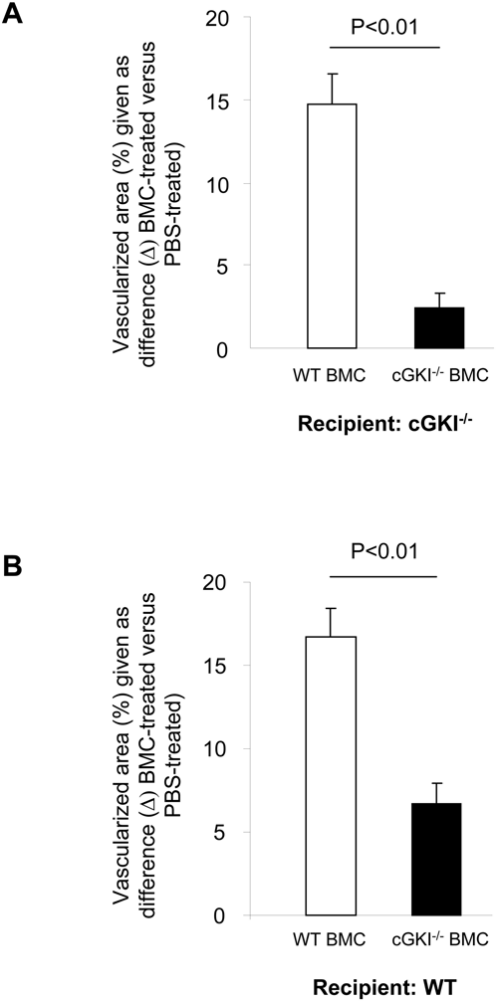
Disc neovascularization following cell therapy. (A) Vascularized area after intravenous injection of cGKI^−/−^ or WT bone marrow cells (BMC) into cGKI^−/−^ recipients (n≥7). (B) Vascularized area after intravenous injection of cGKI^−/−^ or WT bone marrow cells (BMC) into WT recipients (n≥6).

Disc neovascularization depends on both angiogenesis and vasculogenesis. Therefore, sprouting of pre-existing endothelial cells, which have been reported to express cGKI [20], is likely to be involved in disc neovascularization. To evaluate the role of vasculogenesis, i.e. the involvement of bone marrow-derived progenitor cells, in the impaired neovascularization of sponges implanted into cGKI^−/−^ mice, we examined the capacity of bone marrow-derived progenitors to restore neovascularization in the disc model after their systemic infusion. We injected 10^6^ donor cGKI^−/−^ or WT bone marrow mononuclear cells (BMC) into cGKI^−/−^ recipient mice (**Fig. 2A**). Intravenous cell therapy with WT but not cGKI-deficient donor BMC clearly rescued the impaired neovascularization of cGKI^−/−^ recipient mice (p<0.01; **Fig. 2A**). Consistently, neovascularization in WT recipient mice could be strongly enhanced by injection of WT BMC, whereas injection of cGKI^−/−^ BMC had only a weak effect (**Fig. 2B**). These data imply that cGKI in bone marrow progenitor cells plays an important role to restore postnatal neovascularization in an inflammation-induced neovascularization model in cGKI^−/−^ mice.

**Figure 3:**
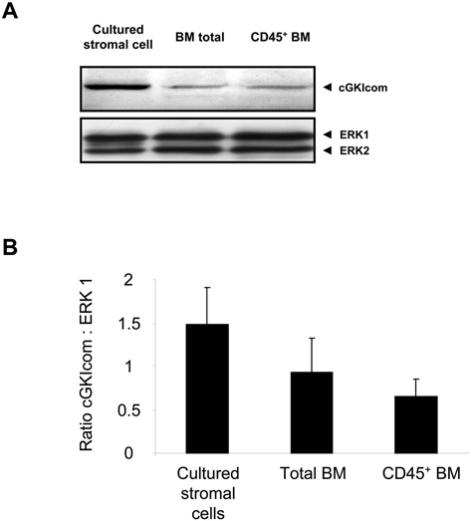
Expression of cGKI in the bone marrow. (A) Representative western blot of cultured bone marrow stromal cells, freshly isolated total bone marrow cells, and freshly isolated CD45^+^ bone marrow leukocytes using an antibody against cGKI (cGKIcom: detecting cGKIα and cGKIβ). ERK1/2 was used as loading control. (B) Quantification of the ratio cGKIcom : ERK1 protein expression in cultured stromal cells, total bone marrow, and CD45^+^ bone marrow in 129/Sv WT mice (n = 3 / group, p = n.s.).

**Figure 4:**
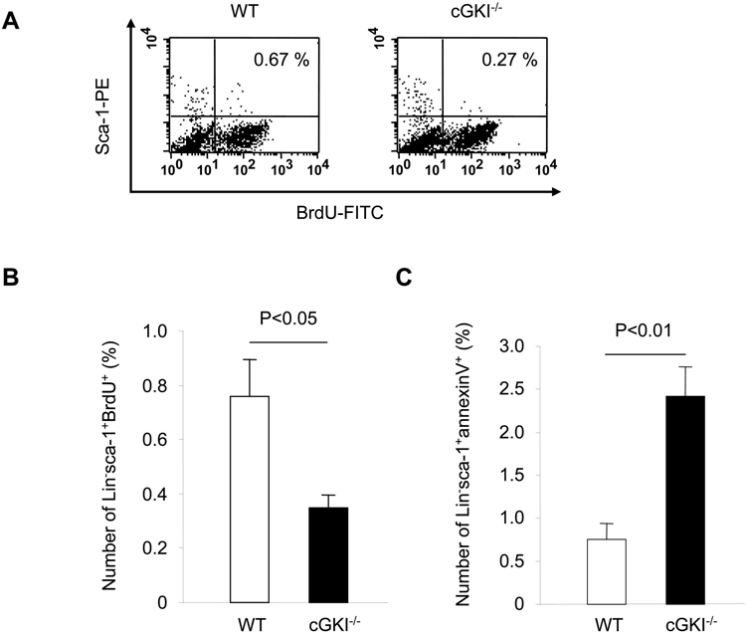
Functional capacity of BMC from cGKI^−/−^ and WT mice. (A) Representative FACS analysis of Lin^−^ BMC after pulsing with BrdU for 1 h and staining with BrdU-FITC and sca-1-PE. (B) Proliferation (BrdU^+^) of sca-1^+^Lin^−^ bone marrow progenitor cells from cGKI^−/−^ and WT mice (gated on lymphocyte-monocyte fraction; n = 4). (C) Apoptosis (annexinV^+^) of sca-1^+^Lin^−^ bone marrow progenitor cells from cGKI^−/−^ and WT mice (gated on lymphocyte-monocyte fraction; n = 3).

To provide further insights into the regulation of vasculogenesis through cGMP-dependent pathways, we examined the role of cGKI on the functional capacity of bone marrow-derived progenitor cells. First, we demonstrated that cGKI is expressed in freshly isolated CD45^+^ hematopoietic bone marrow cells, freshly isolated total bone marrow, as well as cultured bone marrow stromal cells (**Fig. 3A+B**). The most prominent expression of cGKI was found in cultured bone marrow stromal cells, most likely due the relative enrichment of endothelial cells and fibroblasts in cultured bone marrow stromal cells. To assess the functional capacity of bone marrow-derived progenitor cells, we measured proliferation and apoptosis rates in Lin^−^sca-1^+^ bone marrow progenitor cells (**Fig. 4A–C**). BrdU incorporation as a marker of cell proliferation was assessed by flow cytometry and demonstrated a strong reduction in Lin^−^sca-1^+^ bone marrow progenitor cells from cGKI^−/−^ mice (**Fig. 4A+B**). Simultaneously, the number of apoptotic Lin^−^sca-1^+^ bone marrow progenitor cells was increased in cGKI^−/−^ mice as assessed by annexin V binding (**Fig. 4C**). These results suggest that cGKI promotes proliferation and survival of bone marrow-derived progenitor cells, which contributes to neovascularization.

**Figure 5:**
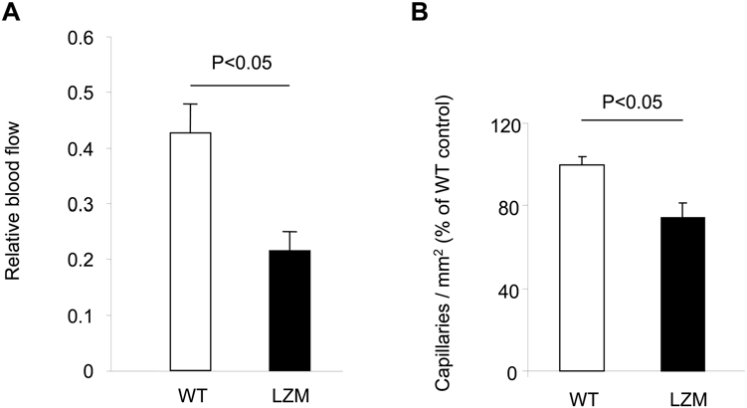
Neovascularization capacity in an unilateral hindlimb ischemia model using LZM and WT mice. (A) Perfusion is measured as relative Laser Doppler-derived blood flow (n≥5). (B) Number of CD31^+^ capillaries / mm^2^ in ischemic hindlimbs of LZM versus WT littermates mice (n = 12–18 sections from 4–6 mice / group).

**Figure 6:**
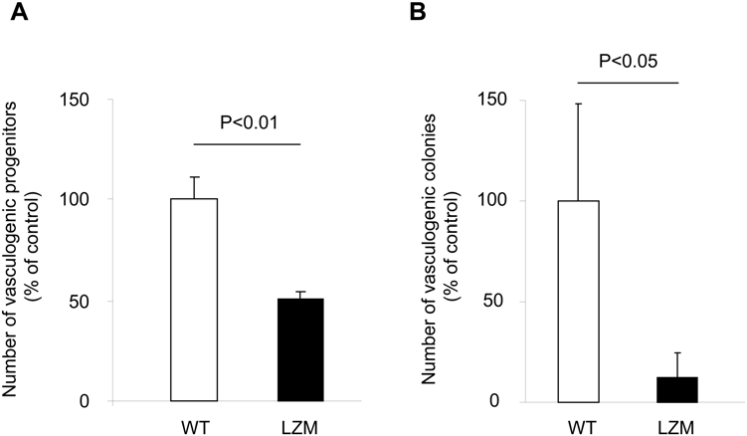
Number of spleen-derived early vasculogenic progenitor cells (A) and vasculogenic colonies (B) in LZM and WT mice (n≥9).

The cGKI^−/−^ mice used for the above experiments show multiple phenotypes and a reduced life expectancy, which complicates long-term experiments with adult mice and excludes assessment of neovascularization in ischemia models [18], [19]. To validate the results obtained in juvenile cGKI^−/−^ mice in an adult model of cGKI deficiency, we also examined the neovascularization capacity of so-called LZM mice [21]. LZM mice carry a mutation in the NH_2_-terminal protein interaction domain of cGKIα that results in disruption of cGKIα interactions with key downstream signaling molecules like myosin phosphatase and Rho kinase, but does not reduce the life span of the mutant mice. In line with the results from juvenile cGKI^−/−^ mice, we observed in LZM mice a reduced neovascularization capacity, measured as relative Laser Doppler-derived blood flow, in a model of hindlimb ischemia (**Fig. 5A**). In addition, the vessel density assessed as number of CD31^+^ capillaries was lower in LZM mice (**Fig. 5B**). Consistently, the number of spleen-derived vasculogenic progenitor cells and vasculogenic colonies was also decreased in LZM mice (**Fig. 6A+B**). The reduced number and colony formation of vasculogenic progenitors might be involved in the impaired neovascularization of LZM mice. These results suggest that cGKIα in vasculogenic progenitors is also involved in the neovascularization response following ischemia.

## Discussion

The major finding of our investigation is a novel role for cGKI in the functionality of bone marrow-derived progenitors. Signaling through cGKI significantly contributed to postnatal neovascularization via stimulation of vasculogenic processes both in the setting of inflammation and following tissue ischemia. Our data implicate that the cGMP-cGKI pathway plays a pivotal stimulatory role for neovascularization, which is at least in part mediated through bone marrow progenitors. cGKI^−/−^ mice exhibit a markedly reduced life span with defects in the relaxation of vascular and visceral smooth muscle cells, disturbed platelets and red blood cells, which limits the availability of adult mice [22]–[25]. Therefore, we further validated our findings using mice with a mutation in the NH_2_-terminal protein interaction domain of cGKIα, the so-called leucine zipper mutant (LZM) mice. This mutation selectively inhibits downstream signaling of the cGKIα isoform while leaving the enzyme intact. LZM mice exhibit a vascular phenotype with increased blood pressure, but reach adulthood without limitations in their life span [21]. Consistently, LZM mice exhibited reduced numbers of vasculogenic progenitors and impaired neovascularization following hindlimb ischemia compared to WT mice.

Several previous studies have analyzed the importance of cGMP signaling for neovascularization, particularly for angiogenesis, i.e. the generation of new vessels through local expansion of mature endothelial cells. For instance, systemic cGMP enhancement augmented angiogenesis in ischemic rat brains [26]. Recently, sildenafil, a phosphodiesterase-5 inhibitor, which inhibits cGMP degradation, has been reported to enhance angiogenesis [27]. Moreover, in a mouse model of hindlimb ischemia, sildenafil promoted recovery of vascular perfusion [28]. Based on the use of pharmacological tools it appeared that neovessel growth in this experimental set up was stimulated by a cGMP-cGK pathway that was independent of nitric oxide production. In line with these previous and our present experiments, a study by Yamahara et al. demonstrated the significance of the the natriuretic peptide-cGMP-cGKI pathway for angiogenesis [29]. However, the involvement of cGMP-cGKI signaling in vasculogenesis, i.e. the contribution of bone marrow-derived progenitor cells to postnatal neovascularization, had not been investigated to date. Our results provide evidence that the reduced neovascularization in cGKI^−/−^ mice is at least in part related to an impaired functional activity of bone marrow-derived progenitors as evidenced by their diminished proliferation and survival, translating into reduced disc neovascularization *in vivo*. The first description that cGMP stimulation could trigger proliferation of hematopoietic stem cells was provided by Oshita et al. back in 1977 [30]. It is now becoming apparent that the cGMP-cGKI signaling pathway has anti-apoptotic/pro-survival effects in diverse cell types [25], [31]–[34].

About 50% of the cGKI^−/−^ mice die before the age of 5 to 6 weeks [18] representing an important drawback of this model. Therefore, we further corroborated our experimental findings derived from cGKI^−/−^ mice using LZM mice. The apparent spontaneous phenotype of LZM mice is hypertension and vascular contractile dysfunction in both large and resistance arteries, but otherwise these mice appear to develop and age normally. More than ten downstream *in vivo* target substrates for cGKI have been found to date [19]. Currently, however, we have not yet identified the downstream target molecules, which might be affected by the disturbed leucine zipper interactions in bone marrow-derived progenitors.

In vascular smooth muscle cells, cGKI binds to myosin light chain phosphatase (MLCP), which mediates relaxation through a leucine zipper domain-mediated interaction [35]. LZM knockin mice harbor a mutation that disrupts the leucine zipper domain required for cGKIα-mediated regulation of MLCP-induced relaxation [21]. Of note, the level of the mutant cGKIα protein in the vascular wall is comparable to the cGKIα protein level found in WT mice, and mutant cGKIα protein remains responsive to cGMP. Thus, key feature of the LZM mouse model is disruption of interactions between cGKIα and its targets, despite preserved kinase activity.

Collectively, our data highlight the *in vivo* importance of the cGMP-cGKI pathway for postnatal neovascularization and particularly emphasize the role of the cGKIα isoform in vasculogenesis. Interestingly, a very recent study demonstrated the potential of the cGMP-elevating drug sildenafil to increase the number of circulating vasculogenic progenitor cells in patients with pulmonary arterial hypertension [36]. Further studies using pharmacological cGMP elevation for the pre-treatment of bone marrow-derived progenitors will give more insights into the therapeutic use of modulating the cGMP-cGKI pathway for cardiovascular cell therapy approaches.

## Materials and Methods

### Animals

The generation of a cGKI null allele (also termed L- allele) by Cre-mediated excision of the loxP-flanked exon 10 of the cGKI gene in germ cells has been described previously [37]. Juvenile (3–5 weeks old) male and female cGKI^−/−^ mice on a 129/Sv genetic background and their wild-type (WT) littermates were used for experiments. Mice with a selective mutation in the NH_2_-terminal protein interaction domain of cGKIα (LZM mice) have been used at adult age (males, C57BL/6 genetic background, crossed >10 generations, 8–10 weeks old) [21]. LZM mice harbor a discrete mutation in cGKIα that disrupts the leucine zipper (LZ) domain that mediates the interaction of cGKIα with downstream target proteins. In LZM mice the initial four leucine/isoleucine codons in exon1a of the endogenous cGKIα gene were replaced by alanines.

For all invasive procedures, mice were anesthetized with ketamine (100 mg/kg). For induction of hindlimb ischemia, anaesthesia was performed using medetomidine (Domitor™; Pfizer, Karlsruhe, Germany; 0.1 mg/kg). For post-operative analgesia carprofen (Rimadyl™; Pfizer; 5 mg/kg) was administered. All animal protocols were approved by the Institutional Animal Care and Use Committee (Regierungspräsidium Darmstadt, Germany).

### BrdU proliferation assay

Total bone marrow cells were obtained by flushing femur and tibia bones with PBS using 27-gauge needles. Then, cells (10^6^ / ml) were resuspended in RPMI 1640 medium+10% fetal calf serum (both from Invitrogen, Karlsruhe, Germany), and pulsed with 10 µM BrdU (BrdU flow kit, BD biosciences (BD), Heidelberg, Germany) for one hour. Staining of surface antigens with a cocktail of biotinylated antibodies against lineage markers (lineage marker panel: anti-CD3, anti-B220, anti-CD11b, anti-Gr, anti-TER119) was performed for 20 min on ice. This step was followed by streptavidin-APC (BD) together with sca-1-PE (BD) staining for another 20 min on ice. Cells were fixed and permeabilized with Cytofix / Cytoperm buffer according to the manufacturer's instructions (BrdU flow kit) and treated with DNase prior to intracellular staining with FITC-labeled BrdU for 20 min at room temperature. Stained cells were then analyzed by flow cytometry using a FACS Calibur (BD) using CellQuest software. Specific cell fluorescence intensity was calculated by subtracting the signal obtained with isotype-matched control antibodies (BD).

### Apoptosis assay

Total bone marrow cells were cultured for 24 h in ex-vivo 10 medium+100 ng/ml murine SCF (Peprotech, Rocky Hill, NJ). Then, bone marrow cells were stained with the biotinylated lineage marker panel (BD) for 20 min on ice, followed by streptavidin-PE (BD) together with sca-1-FITC and annexinV-APC (both BD) for another 20 min at room temperature using annexin binding buffer (BD). Cells were then immediately analyzed by flow cytometry using a FACS Calibur.

### Vasculogenic progenitor assay

Spleens were aseptically removed, homogenized, and laid over Ficoll solution (Biocoll, Biochrom AG, Berlin, Germany). Mononuclear cells were obtained from the Ficoll's interphases and washed with PBS. Then, 4×10^6^ cells per well were seeded on fibronectin-precoated 24-well plates (10 µg/ml fibronectin in PBS for 1 h at room temperature; Sigma-Aldrich, Taufkirchen, Germany) and cultured in 500 µl EBM medium+20% FCS+supplements (Cambrex Bio Science, Verviers, Belgium) at 37°C. After four days of culture, vasculogenic progenitors were assayed by co-staining with 1,1′–dioctadecyl–3,3,3′,3′–tetramethylindocarbocyanine-labeled acetylated low-density lipoprotein (DiLDL; CellSystems, St. Katharinen, Germany) and FITC-conjugated lectin (Sigma-Aldrich) [38].

To obtain colonies with vasculogenic activity, we used a modified version of the protocol of Ingram et al. [39] using fibronectin and supplemented EBM medium scoring clusters of cells after 7 days.

### Western blot analysis

Total bone marrow cells, CD45^+^ bone marrow leukocytes, and cultured bone marrow stromal cells were used for Western blot analysis. CD45^+^ cells were isolated by immunomagnetic selection using magnetically labeled anti-CD45 microbeads and magnetic cell sorting (Miltenyi Biotec, Bergisch Gladbach, Germany). To obtain bone marrow stromal cells, total bone marrow cells were cultured in RPMI 1640 medium+10% fetal calf serum (both from Invitrogen) for one week. Cells were fed with fresh medium every other day and non-adherent cells were discarded. After trypsinization of the adherent stromal cells, the pelleted cells were resuspended in lyzing buffer. Finally, proteins were separated on a SDS gel, transferred to a PVDF membrane and stained with a polyclonal rabbit antiserum to cGKI [40]. ERK1/2 (p44/p42 MAPK) antibodies were used as a loading control (Cell Signaling Technology, Danvers, MA).

### Disc neovascularization model

A disc of polyvinyl alcohol sponge (Rippey Corp., El Dorado Hills, California), covered with nitrocellulose cell-impermeable filters (Millipore Corp., Burlington, Massachusetts), allows vessels to grow only through the rim of the disc [41], [42]. The discs were subcutaneously implanted in the back of 3–5 weeks old cGKI^−/−^ mice or their WT littermates. For cell therapy, bone marrow cells were harvested from donor cGKI^−/−^ or WT mice by aseptically flushing femurs and tibias with PBS. Bone marrow mononuclear cells (BMC) were isolated by density-gradient centrifugation with Biocoll separating solution (density 1.077; Biochrom AG). Twenty-four hours after implantation of sponges, we intravenously injected 10^6^ BMC from either cGKI^−/−^ or WT BMC . Two weeks later, space-filling fluorescent microspheres (0.2 µm; Molecular Probes Inc., Eugene, OR) were systemically injected into the left ventricle to identify functionally connected microvessels in the implaned disks [43]. The area of the disc invested by fibrovascular ingrowth was assessed using a Axiovert 40 fluorescence microscope (Zeiss, Oberkochen, Germany). Alternatively, outgrowth of vessels was examined by CD31 staining (CD31-FITC; BD biosciences, Heidelberg, Germany) for endothelial cells.

### Hind limb ischemia model

To induce unilateral hind limb ischemia, the proximal portion of the right femoral artery including the superficial and the deep branch were occluded using an electrical coagulator. The overlying skin was closed using surgical staples. Limb perfusion was assessed with a laser Doppler blood flow imager (Laser Doppler Perfusion Imager System, moorLDI™-Mark 2, Moor Instruments, Wilmington, Delaware) after one week. Before initiating scanning, animals were placed on a heating pad at 37°C to minimize variations in temperature. Calculated perfusion is expressed as the ratio of ischemic to non-ischemic hind limb perfusion.

### Histology

Capillaries were scored in 8 µm frozen sections of the adductor muscles by staining for CD31 (PE-labeled; BD Biosciences). Images were obtained by confocal microscopy (LSM 510 Meta, Zeiss, Germany).

### Statistical analysis

Results are expressed as mean±SEM. Overall comparison of the treatment groups was performed with the Kruskal-Wallis test followed by post-hoc pairwise comparison using the Mann-Whitney test. P values<0.05 were considered statistically significant. Analyses were performed with SPSS 15.0 (SPSS Inc.).
